# Ubiquitination and Deubiquitination in Oral Disease

**DOI:** 10.3390/ijms22115488

**Published:** 2021-05-23

**Authors:** Sachio Tsuchida, Tomohiro Nakayama

**Affiliations:** Divisions of Laboratory Medicine, Department of Pathology and Microbiology, Nihon University School of Medicine, Tokyo 173-8610, Japan; nakayama.tomohiro@nihon-u.ac.jp

**Keywords:** Ubiquitination, deubiquitination, deubiquitinating enzymes, ubiquitin-conjugating enzyme, oral disease

## Abstract

Oral health is an integral part of the general health and well-being of individuals. The presence of oral disease is potentially indicative of a number of systemic diseases and may contribute to their early diagnosis and treatment. The ubiquitin (Ub) system has been shown to play a role in cellular immune response, cellular development, and programmed cell death. Ubiquitination is a post-translational modification that occurs in eukaryotes. Its mechanism involves a number of factors, including Ub-activating enzymes, Ub-conjugating enzymes, and Ub protein ligases. Deubiquitinating enzymes, which are proteases that reversely modify proteins by removing Ub or Ub-like molecules or remodeling Ub chains on target proteins, have recently been regarded as crucial regulators of ubiquitination-mediated degradation and are known to significantly affect cellular pathways, a number of biological processes, DNA damage response, and DNA repair pathways. Research has increasingly shown evidence of the relationship between ubiquitination, deubiquitination, and oral disease. This review investigates recent progress in discoveries in diseased oral sites and discusses the roles of ubiquitination and deubiquitination in oral disease.

## 1. Introduction

Oral health is essential to general health and well-being. The presence of oral disease may contribute to the early diagnosis and treatment of a number of systemic diseases, and the oral cavity is an integral component of the relationship between oral and systemic health [1,2].

The ubiquitin (Ub) system has been implicated in cell-mediated immune responses, cellular development, and programmed cell death. In recent years, the Ub–proteasome system (UPS) and autophagy have reportedly been closely associated with such pathological conditions as neurodegenerative diseases, cancer, aging, and metabolic disorders. Post-translational modification ubiquitination is restricted to eukaryotes that participate. It is achieved by a mechanism that involves several factors, including Ub-activating enzymes, Ub-conjugating enzymes, and Ub protein ligases.

Initially, UPS considered responsible for the facilitation of the degradation of old proteins—that is, cleaning up after cellular activities. It is now known that UPS plays an important role in cell proliferation and differentiation by controlling quantitative changes in cell cycle–related factors, such as signal transduction and transcription factors [3,4,5,6,7,8,9]. It is also closely involved in a wide range of immunological processes, such as antigen processing and protein quality control mechanisms in the endoplasmic reticulum. This latter process may be involved in immunological processes, but plays a much broader role.

Deubiquitinating enzymes (DUBs) are proteases that reverse protein ubiquitination, a process that is important in normal homeostasis, or trim Ub chains of diverse linkages [10,11,12]. The variety of cellular processes initiated and regulated by ubiquitin has been explained in part by the structural diversity of differently linked ubiquitin chains [13].

DUBs have four distinct mechanisms of action: (1) processing of Ub precursors, (2) recycling of Ub molecules during ubiquitination, (3) cleavage of poly-Ub chains, and (4) reversal of Ub conjugation. They regulate several cellular functions, including proteasome- and lysosome-dependent proteolysis, gene expression, cell cycle progression, and chromosome segregation [14]. DUBs also exhibit various catalytic mechanisms. They can unknot Ub conjugation by cleaving the bond between Ub molecules and Ub–target complexes, editing Ub chains to remove one or more Ub molecules, and recycling Ub molecules in the Ub–proteasome pathway. Furthermore, they are involved in kinase activation, apoptosis, localization, DNA repair, and maintenance of stemness. Most of these processes involve non-degradative Ub chains [15,16,17,18,19,20]. The ubiquitin modification system is a post-translational modification system in which ubiquitin, a low-molecular-weight globular protein of 8.6 kDa, is isopeptidically added to the Lys residue side chain of a target protein via the activities of three enzymes: E1 (ubiquitin-activating enzyme), E2 (ubiquitin-binding enzyme), and E3 (ubiquitin ligase) [21,22]. There are two types of E1 in humans. In humans, there are two types of E1, approximately 50 types of E2 and approximately 600 types of E3; furthermore, E3 plays the most crucial role in identifying the target protein to be ubiquitinated in a spatiotemporally specific manner. The ubiquitin bound to the target protein is removed and reused by nearly 100 different deubiquitinating enzymes.

Ubiquitinating enzymes (UBEs) catalyze protein ubiquitination, a reversible process countered by DUB action [20,23]. UBEs consist of three structurally and functionally different classes of enzymes, namely E1, E2, and E3. Humans carry two E1 genes, whereas yeast carries only one. UBEs catalyze the covalent addition of ubiquitin, which is a 76-amino acid molecule, to target proteins via the sequential action of a ubiquitin-activating enzyme (E1) and a ubiquitin-conjugating enzyme (E2) that selectively interacts with a ubiquitin ligase (E3) to control substrate specificity [24].

To understand the non-degradative roles of ubiquitination is very important [25,26]. Reportedly, non-degradative ubiquitination in signaling by innate receptors, particularly the TLRs, but also in response to the pro-inflammatory cytokine interleukin-1, which, similar to TLRs, signals via the Toll/interleukin-1 receptor domain [27]. Furthermore, it is unclear how polyubiquitination is a key activation signal for the transcription factor NF-kB.

Although the critical role of ubiquitination in the proteasomal degradation of target proteins is well known, the non-degradative roles of ubiquitination have not yet been described thoroughly in the literature [28]. Moreover, existence of different types of ubiquitin chains is fundamental. Ubiquitin chains are made by linking the glycine residue of a ubiquitin molecule to a lysine of ubiquitin bound to a substrate. Ubiquitin has seven lysine residues and an N-terminus that serves as ubiquitination points: K6, K11, K27, K29, K33, K48, K63, and M1, respectively [29]. Lysine 48-linked chains were the first identified and are the best-characterized type of ubiquitin chain. K63 chains have been characterized.

Considering that evidence of the relationship between ubiquitination, deubiquitination, and oral disease is growing, this review aimed to tackle emerging data on ubiquitination and deubiquitination given that they are associated with oral diseases.

## 2. Types of Dental and Oral Diseases

Three mechanisms or pathways linking oral infections to secondary systemic effects have been proposed: (i) metastatic spread of infection from the oral cavity as a result of transient bacteremia; (ii) metastatic injury from the effects of circulating oral microbial toxins; and (iii) metastatic inflammation caused by immunological injury induced by oral microorganisms. Oral bacteria and the inflammation associated with a severe form of periodontitis might play a role in some diseases [30]. In addition, certain diseases, such as diabetes and HIV/AIDS, can lower the body’s resistance to infection, thereby exacerbating the severity of oral health problems.

### 2.1. Cavities

Cavities are permanently damaged surface areas on a tooth where miniscule holes begin to appear and thereby expose the tooth or make it susceptible to further bacterial accumulation or decay [31,32]. In C1 (where “C” stands for caries), the decay in its early stages is confined to the enamel, i.e., only the surface of the dentin is affected. In C2, tooth decay progresses into the dentin but does not affect the nerve of the tooth. In C3, the cavity progresses to the nerve of the tooth, which becomes inflamed as a result of bacterial infection. C4 is considered the final stage of tooth decay, where only the root remains.

### 2.2. Gingivitis

Gingivitis is prevalent across all ages of the dentate population and is considered to be the most common form of periodontal disease [33,34,35]. It is characterized by inflammation of the gums due to excessive plaque on the teeth without attachment loss [36]. The causes of plaque-induced gingivitis may differ from those of non-plaque-induced gingivitis: Plaque-induced gingivitis is characterized by inflammation of the gingiva caused by bacteria located at the gingival margin, whereas non-plaque-induced gingivitis often exhibits characteristic clinical features and can be caused by allergic reactions to such stimuli as dental restorative materials, toothpastes, mouthwashes, and food.

Plaque, or clumps of bacteria, between teeth and that on the border between the teeth and gums cause symptoms of inflammation only on the gums, which would initially turn slightly bloodshot and red and then become swollen. Brushing will cause bleeding, but the bone that supports the teeth will remain intact.

### 2.3. Periodontal Diseases

Bacterial infections in the periodontium lead to periodontal diseases, which consequently result in the loss of tooth support and are associated with bacteria-induced inflammation and activation of the host immune response [37,38]. Periodontitis occurs in three stages: mild, moderate, and severe. Mild periodontitis is the earliest stage of the disease, and it occurs when plaque begins to harden into calculus between the gums and teeth. Some systemic diseases have been linked to periodontal health as an exacerbation or manifestation of the primary disease process. The current classification of periodontal disease includes four stages which are based on disease severity and complexity of management [39]. These categories include: Stage 1 (initial periodontitis), Stage 2 (moderate periodontitis), Stage 3 (severe periodontitis with potential for additional tooth loss), and Stage 4 (severe periodontitis with potential for loss of dentition). Periodontal disease is also classified by its extent and distribution, including localized, generalized, or molar–incisor distribution. Finally, the disease is categorized by grade based on the evidence of risk of rapid progression and anticipated treatment responses, the grades include Grade A (slow rate of progression), Grade B (moderate rate of progression), and Grade C (rapid rate of progression). Consequently, the toxic substances produced by inflammation can enter the entire body through the blood vessels in the gums and thereby cause or aggravate various diseases, such as diabetes, premature birth, low birth weight, obesity, and arteriosclerosis.

The occurrence of periodontal disease indicates constant inflammation in the mouth. Inflammatory substances are also involved in worsening the function of insulin to lower blood sugar levels (diabetes), premature birth, low birth weight, obesity, and arteriosclerosis of blood vessels (myocardial and cerebral infarctions).

Bacterial pathogens are strongly associated with the progression of periodontal disease [40]. Some of the aspects of microbial dental plaques [41], i.e., development and heterogeneity, microbial succession, composition, structure, mechanisms of formation, need to be explored so that the microbial composition of the gingival crevice (subgingival plaque) could be understood further. Microbial dental plaques may be classified as heterogeneous, dense, non-calcified bacterial masses that are typically firmly adhered to the tooth surface such that the flow of saliva does not wash them off. Some subgingival plaques are nonadherent and consist of several motile organisms [42,43].

The following species, which are referred to as red complexes, are strongly associated with the onset and progression of periodontal disease: (1) *Porphyromonas gingivalis*: *P. gingivalis* is difficult to detect beyond the affected area of periodontal disease, and it is considered to significantly affect periodontal inflammation and other symptoms of periodontal disease. (2) *Treponema denticola*: *T. denticola* contains proteins that settle in periodontal tissues; it is often found in subgingival plaque. It destroys periodontal tissues via proteolytic enzymes and has been suggested to interfere with healing by suppressing the immune system. (3) *Tannerella forsythensis*: *T. forsythensis* is used as an indicator of intractable periodontal disease that is not easily cured by standard periodontal treatment.

In addition, *Aggregatibacter actinomycetemcomitans* has been widely detected in juvenile periodontitis. Furthermore, *Fusobacterium nucleatum*, which is indigenous to the human oral cavity, is considered to be central in plaque (biofilm) formation.

### 2.4. Cracked or Broken Teeth

A cracked or broken tooth can cause pain and embarrassment and affect mastication. It involves either an incomplete fracture that begins on the surface of the enamel/root and terminates within the tooth or a complete fracture that separates a portion of the crown where the fracture occurs horizontally or results in a split tooth where the fracture occurs vertically [44,45,46]. Cracked/broken teeth can be caused by untreated cavities, trauma, vehicular accidents, contact sports, falling, biting on hard foods (e.g., ice, nuts, and seeds), using teeth to open bags or packages, and excessive grinding or clenching of teeth. Nevertheless, these conditions are treatable. Cracked and broken teeth are also presumed to cause inflammation (NF-κB), which would consequently be regulated by ubiquitination.

### 2.5. Tooth Sensitivity

Tooth sensitivity, or dentin hypersensitivity, occurs when the root dentin surface becomes exposed and reacts to activities such as eating, drinking, chewing, and brushing [47,48,49]. Dentin contains fluid-filled microscopic tubules (rods) that trigger a response often described as a painful zing when exposed to a stimulus. Opening of dentin tubules, typically due to gingival recession or enamel loss, allows them to come in contact with irritants, which in turn can consequently induce fluid movement within them. This fluid movement may stimulate the nerves in the tubules, and thereby trigger a sudden, sharp tooth pain. Currently, cracked or broken teeth and perhaps tooth sensitivity has been linked to ubiquitination and deubiquitination activity.

### 2.6. Oral Cancers

Oral cancer refers to any cancer occurring in the front area of the mouth, including the lips, tongue, inner surface of the cheeks, hard palate (the front of the roof of the mouth), and gums [50,51,52,53,54,55]. Alternatively, throat cancer refers to any cancer affecting the back part of the mouth, including the soft palate or the back of the throat. Oral squamous cell carcinoma (OSCC) is a type of oral cancer in which surface cells grow and divide in an uncontrolled manner [56,57]. Early detection and prevention of oral cancer are crucial as delayed diagnosis is considered to be one of the primary reasons for the low 5-year survival rate of patients, thereby increasing the demand for oral cancer screening. Currently, screening for oral cancer is largely performed by visual examination. Several lines of evidence strongly highlight the validity of visual inspection in reducing mortality in patients at risk for oral cancer. A visual examination, accompanied by adjunctive techniques for subjective interpretation of dysplastic changes, includes toluidine blue staining, brush biopsy, chemiluminescence, and tissue autofluorescence. The risk factors for oral cancer include tobacco smoking and alcohol use.

## 3. Ubiquitination and Deubiquitination in Oral Disease

Understanding of the molecular action of Ub in signaling pathways is expanding, and the effects of alterations in DUBs and the Ub system on the development of oral disease are being investigated (Supplementary Table S1). Advances of technology in the field of proteomics using mass spectrometry combined with the development of specific antibodies against Ub chains or Ub remnants on substrates allow for precise tracking of ubiquitination throughout the proteome. Therefore, novel Ub-regulatory enzymes, as well as DUBs and adaptors, were identified as possible targets in the development of more selective therapeutic compounds for the treatment of oral disease [58,59].

The Ub system plays a significant role in regulating cellular proliferation [60]. Alterations in specific pathways involving Ub and DUBs have been linked to oral cancer. The stability of the transcription factor p53, which has a crucial role in cellular anticancer mechanisms, is regulated by Ub ligases and deubiquitinating enzyme (DUB). The concept of p53 oncogenic mutation has been proposed. It has been shown that most p53 mutations in oral cancer are oncogenic. Mutations in the p53 gene have been detected in approximately >50% of tumors [61]. Targeting the proteasomal degradation pathway is increasingly getting recognized as a promising strategy for cancer therapy [62,63,64]. The UPS pathway primarily degrades the wild-type (WT) p53 protein. However, mutations in p53 might stabilize this protein and inhibit its interaction with murine double minute 2 (MDM2), a protein that suppressively regulates the activity of p53, a tumor suppressor, thereby preventing degradation [65].

Considering UPS’s central role in protein homeostasis, malfunctions of UPS components result in various diseases, including cancer, developmental disorders, neurodegenerative diseases, and autoimmune diseases. The 26S proteasome is a 2.5-MDa complex that causes selective degradation of ubiquitylated proteins. Cell proliferation is contingent on the proteasome activity, and cancer cells require higher proteasome levels to cope with chronic proteotoxic stress. Accordingly, proteasome inhibitors, such as bortezomib (BTZ) and carfilzomib, are used in cancer therapy method [66,67,68]. The successful development of proteasome inhibitors has piqued interest in exploring the potential of other UPS components as drug targets.

Similar to other post-translational modifications, ubiquitination is a reversible process. Covalently attached Ub molecules can be removed by DUBs, which preserve cellular Ub pools, alter inappropriate ubiquitination, and dynamically regulate processes in which ubiquitination participates [69]. The human genome encodes approximately 100 DUB enzymes belonging to six different families [12,70,71]. Although DUBs primarily antagonize ubiquitination processes, they may occasionally promote them by reversing the auto-ubiquitination of E3 enzymes, such as deubiquitinase herpes virus-associated Ub-specific protease (also known as USP7) and E3 ligase mouse double minute 2 [72].

In protein ubiquitination, E1 and ATP activate Ub. The E1 enzyme subsequently passes the Ub protein on to E2s. The E2s then conjugate with Ub ligase E3s. Lastly, E3s catalyze the transfer of Ub to substrates. Proteins can undergo various types of ubiquitination, including monoubiquitination and polyubiquitination [73], wherein they are degraded in the lysosome and proteasome, respectively. In protein deubiquitination, DUBs remove Ub molecules from target substrates.

The DUB cylindromatosis (CYLD) acts as a tumor suppressor in several malignancies [74,75]. Data have shown that loss of CYLD expression in tissues is significantly associated with poor prognosis in patients with OSCC. Further research has demonstrated that CYLD negatively regulates multiple cell signaling pathways, including nuclear factor-kappaB (NF-κB), Wnt/β-catenin, c-Jun N-terminal kinase (JNK), p38/mitogen-activated protein kinase, and Hippo and Notch signaling [17,76,77,78,79,80,81,82,83,84]. The CYLD gene has been initially identified as a mutation because its loss causes cylindromas, which are benign tumors.

Ub-specific protease 14 (USP14), another DUB, has been implicated in the tumorigenesis and progression of several cancers. Chen et al. demonstrated its expression pattern in OSCC and confirmed its role in tumor growth and metastasis [85]. Such results can help validate the use of USP14 selective inhibitors, such as b-AP15, or knockdown by shRNA in novel targeted cancer therapies and suggest that USP14 is a potential therapeutic target for OSCC [85].

Ubiquitin-specific protease (USP22) promotes stability of multiple cancer-associated protein targets through deubiquitylation (e.g., TRF1 and SIRT1) [86,87], and influences oncogene accumulation [88]. The expression of USP22 increases with the progression of oral carcinogenesis from non-cancerous mucosa to primary carcinoma and from carcinomas to lymph node metastasis. Remarkably, patients with OSCC whose samples showed positive USP22 expression were reported to have significantly poorer outcomes compared with patients whose samples demonstrated negative expression, indicating that OSCC is characterized by the down-modulation, not up-regulation, of Ub-specific peptidase 9, X-linked (USP9x) [89,90]. Liu T et al. suggested that USP22 may be involved in the progression of OSCC, in cooperation with Aurora-B and Survivin, which belong to the chromosomal passenger complex, are also highly expressed in several types of cancer [91]. USP9X is one of the most abundant members of the USP family and has been linked with many processes, including centrosome function, chromosome alignment during mitosis, EGF receptor degradation, chemo-sensitization, and circadian rhythms [92,93,94,95,96,97]. Nanayakkara et al. reported that the deubiquitylating enzyme, USP9X, possibly promotes head and neck cancer cell proliferation through the mTOR pathway [89]. India Project Team of the International Cancer Genome Consortium reported that Exome sequencing and recurrence testing reveals that some significant and frequently altered genes are specific to Gingivo-buccal oral squamous cell carcinoma OSCC-GB (USP9X) [90]. In contrast, some others are shared with head and neck squamous cell carcinoma (HNSCC) [90]. Moreover, USP9x was found to be mutated in a panel of OSCC cell lines [98].

The immune checkpoint protein programmed cell death ligand 1 (PD- L1) binds to PD1 to promote tumor escape from cell death. Jingjing et al. discovered that USP9X [99] could bind to PD-L1 to induce its deubiquitination and stabilize its protein expression in OSCC cells [58].

Research into ubiquitination and its contribution to periodontal disease is progressing steadily. We previously identified the antimicrobial peptide dermcidin (DCD) in the gingival crevicular fluid using proteomic analysis [100]. Furthermore, we examined Ubs among DCD-interacting proteins in detected ubiquitinated DCD using Western blotting as well as immunoprecipitation with antibodies against DCD and mono/polyubiquitinated proteins [101]. These analyses conclude that DCD may be ubiquitinated [101].

Muramyl dipeptide (MDP) is a bacterial cell wall component in both Gram-positive and Gram-negative bacteria. It is composed of *N*-acetylmuramic acid that is linked by its lactic acid moiety to the N-terminus of an l-alanine d-isoglutamine dipeptide. It is transferred into the cytoplasm, where it activates JNKs, which in turn upregulate activator protein 1. Cai et al. [102] reported that MDP highly activates signaling pathways in response to *P. gingivalis* infection. *P. gingivalis*–induced tumor necrosis factor-alpha (TNF-α) expression can be affected by MDP in a biphasic concentration-dependent manner. MDP transferred into the cytoplasm activates JNKs, which subsequently up-regulates activator protein-1. JNKs are essential regulators of physiological and pathological processes in several diseases, whereas activator protein-1 activates the Ub-editing enzyme A20 and restricts ubiquitination of nucleotide-binding oligomerization domain-containing protein 2, inhibiting TNF-α secretion in response to the infection of *P. gingivalis*. A20 has been shown to regulate NF-κB signaling negatively via multiple mechanisms, such as binding of inflammatory molecules, including TNF-α, interleukin (IL)-1β, lipopolysaccharide (LPS), cluster of differentiation 40, and IL-17, to their respective cell surface receptors, which facilitates recruitment of specific adaptor proteins. In addition, it is a potent inhibitor of NF-κB signaling [103] and an immediate–early target gene of NF-κB that is involved in the termination of NF-κB activation as part of a negative feedback loop. NF-κB is activated by inflammation, the immune system, cell stress, and inflammatory cytokines, including IL-1, TNF-α, volvulus esters, lectins, calcium ionophore, LPS, human T cells, leukemia virus Tax protein, hepatitis B virus X protein, and adenovirus EIA.

The activity of deubiquitinylase in the ovarian tumor domain and that of Ub E3 ligase in the fourth zinc finger of A20 play a crucial role in this process [103,104]. A20 also regulates cell death. A20 has regulated autophagy triggered by the LPS receptor of Toll-like receptor 4 [105]. The study reported the effects of A20 Overexpression on the inflammatory response in patients with periodontitis and found that A20 was up-regulated in gingival tissues and neutrophils as well as in LPS-exposed human periodontal ligament cells. Overexpression of A20 is a potential therapeutic target in inflammatory bone loss diseases, including periodontal disease [106].

Cracked or broken tooth is caused by fracturing of the tooth from the biting surface inwards and toward the tooth root. Cases of broken or cracked tooth may cause excruciating toothaches considering that they typically expose the inner pulp of the tooth. Tooth sensitivity can be very uncomfortable and cause avoidance of some foods and beverages. The numerous possible causes for tooth sensitivity include worn tooth enamel and exposed tooth root surface, among others. Shuang Jiang et al. reported the USP34-deficient dental pulp cells (DPCs) exhibit decreased odontogenic differentiation with downregulation of nuclear factor I/C (NFIC) and Overexpression of NFIC partially restores the impaired odontogenic potential of DPCs [107]. They reported that ubiquitin-specific protease 34 (USP34) plays a pivotal role in tooth root formation; findings indicate that USP34-dependent deubiquitination is critical for root morphogenesis by stabilizing NFIC [107].

Saliva is a vital fluid in the maintenance of oral homeostasis, and reduction predisposes individuals to oral symptoms and oral disease. Imamura Y et al. provided further evidence that histatin 3 may be involved in regulating cell proliferation, particularly during G1/S transition, via the ubiquitin-proteasome system of p27(Kip1) and HSC70 [108]. Karbanová J et al. reported investigated its expression in various human salivary gland lesions using two different anti-prominin-1 monoclonal antibodies applied on paraffin-embedded sections and characterized its occurrence in saliva [109]. It is assumed that saliva, and the lack thereof, could indirectly affect ubiquitination and deubiquitination activities.

Currently, proteolysis with mass spectrometry is considered the analytical method of choice for detection studies of ubiquitinated proteins [110]. Notably, Danielsen et al. identified putative 5756 ubiquitinated proteins in U2OS osteosarcoma cells and HEK293T embryonic kidney cells with mass spectrometry [111]. There are two types of malignant tumors that develop in the mandibula: gingival carcinoma and other malignant tumors that arise from the surrounding soft tissues and invade the mandibula, and osteosarcoma, which develops centrally in the mandibula. In the future, it is expected that proteomic analysis will be used to elucidate the ubiquitination of mandibula cells. Anti-ubiquitin antibody-based label-free quantitative proteomics with mass spectrometry is an effective method to globally detect, identify, and quantify protein ubiquitination in a given condition, such as disease, tumors, or health [112,113,114,115].

Notch signaling links to the ubiquitination system [116]. Notch signaling is regulated by ubiquitination of the receptor and its extracellular ligands reveal distinct ubiquitination-dependent endosomal sorting pathways [117]. Notch pathway can be seen as a model system where numerous and different types of ubiquitination events, targeting various substrates, with various types of chains, localizations, and outcomes, are used. Osathanon et al. demonstrated that Notch signaling is dysregulated in human OSCC and plays a role in cell proliferation [118]. Ubiquitination is the main player in regulating various cellular processes, including cell division, differentiation, signal transduction, and protein trafficking. Additionally, ubiquitin, a versatile modification designed to shape cell-signaling pathways, is an important part of the Notch pathway [119]. Further research elaborating on the dysregulation of Notch signaling in OSCC and how it relates to the ubiquitination system would prove to be beneficial.

## 4. Future Directions for Oral Disease

The interaction between ubiquitination and deubiquitination plays crucial roles in almost all aspects of biological activities. This phenomenon is expected to apply to diseases in the future, and understanding of ubiquitination and deubiquitination may provide novel insights into the treatment of oral diseases.

To date, there is a paucity of information on NF-κB activation in oral diseases, periodontal diseases, and oral cancer, thereby highlighting the need for further research. The Ub-proteasome pathway is a therapeutic target for such hematological malignancies as multiple myeloma and non-Hodgkin lymphoma. Laboratory studies are evaluating the clinical application of BTZ, a first-generation proteasome inhibitor, as a potential therapeutic agent for these diseases. Its potential efficacy in the treatment of oral disease should likewise be explored.

A ubiquitinated protein enrichment kit has recently been developed. This kit aims to enrich ubiquitinated proteins from cell/tissue lysates via immunoprecipitation using affinity beads [120,121]. After ubiquitinated proteins are concentrated, they are analyzed by Western blotting using primary antibodies (not included in the kit) that recognize the protein of interest. This facilitates the examination of transient regulatory mechanisms, measurement of multiple proteins involved in signaling pathways, and identification of new modifications of target proteins. Therefore, the analysis of regulatory mechanisms, such as ubiquitination and deubiquitination in oral disease, is becoming increasingly achievable.

## 5. Conclusions

This review evaluated the existing literature on ubiquitination and deubiquitination in oral disease. Research on DUBs is crucial to elucidating the role of the UPS pathway in various diseases. Recent advances in the field of cellular biology and proteasomal pathways provide an optimistic outlook toward new and more effective treatments for many illnesses. Identifying the underlying mechanisms of DUBs and ubiquitination-associated oral diseases will help expand current knowledge about their relationship. Oral diseases constitute a significant public health problem worldwide, and individual circumstances as well as the level of access to certain resources and opportunities play a role in their development. In clinical practice, we are often made to think about the effects of systemic diseases on the oral cavity. Systemic diseases that affect the oral cavity include diabetes, obesity, visceral fat syndrome (metabolic syndrome), osteoporosis, and immune system diseases. However, in addition to these, factors such as hormonal imbalance, smoking, and stress are also deeply related to oral diseases, especially periodontal diseases. The potential correlation between systemic diseases and typical oral clinical conditions, which significantly affect a patient’s oral health, should thus be further investigated with emphasis on ubiquitination and deubiquitination in oral disease.

## Supplementary Materials

The following are available online at https://www.mdpi.com/article/10.3390/ijms22115488/s1.
